# Skeletal muscle endurance declines with impaired mitochondrial respiration and inadequate supply of acetyl-CoA during muscle fatigue in 5/6 nephrectomized rats

**DOI:** 10.1152/japplphysiol.00226.2023

**Published:** 2023-08-10

**Authors:** Hiroyori Fusagawa, Tatsuya Sato, Takashi Yamada, Yuki Ashida, Iori Kimura, Azuma Naito, Nao Tokuda, Nao Yamauchi, Nobutoshi Ichise, Yoshinori Terashima, Izaya Ogon, Atsushi Teramoto, Toshihiko Yamashita, Noritsugu Tohse

**Affiliations:** ^1^Department of Cellular Physiology and Signal Transduction, Sapporo Medical University School of Medicine, Sapporo, Japan; ^2^Department of Orthopaedic Surgery, Sapporo Medical University School of Medicine, Sapporo, Japan; ^3^Department of Cardiovascular, Renal and Metabolic Medicine, Sapporo Medical University School of Medicine, Sapporo, Japan; ^4^Graduate School of Health Sciences, Sapporo Medical University, Sapporo, Japan

**Keywords:** cachexia, chronic kidney disease, metabolome, mitochondria, muscle fatigue

## Abstract

Chronic kidney disease (CKD)-related cachexia increases the risks of reduced physical activity and mortality. However, the physiological phenotype of skeletal muscle fatigue and changes in intramuscular metabolites during muscle fatigue in CKD-related cachexia remain unclear. In the present study, we performed detailed muscle physiological evaluation, analysis of mitochondrial function, and comprehensive analysis of metabolic changes before and after muscle fatigue in a 5/6 nephrectomized rat model of CKD. Wistar rats were randomized to a sham-operation (Sham) group that served as a control group or a 5/6 nephrectomy (Nx) group. Eight weeks after the operation, in situ torque and force measurements in plantar flexor muscles in Nx rats using electrical stimulation revealed a significant decrease in muscle endurance during subacute phase related to mitochondrial function. Muscle mass was reduced without changes in the proportions of fiber type-specific myosin heavy chain isoforms in Nx rats. Pyruvate-malate-driven state 3 respiration in isolated mitochondria was impaired in Nx rats. Protein expression levels of mitochondrial respiratory chain complexes III and V were decreased in Nx rats. Metabolome analysis revealed that the increased supply of acetyl CoA in response to fatigue was blunted in Nx rats. These findings suggest that CKD deteriorates skeletal muscle endurance in association with mitochondrial dysfunction and inadequate supply of acetyl-CoA during muscle fatigue.

**NEW & NOTEWORTHY** Mitochondrial dysfunction is associated with decreased skeletal muscle endurance in chronic kidney disease (CKD), but the muscle physiological phenotype and major changes in intramuscular metabolites during muscle fatigue in CKD-related cachexia remain unclear. By using a 5/6 nephrectomized CKD rat model, the present study revealed that CKD is associated with reduced tetanic force in response to repetitive stimuli in a subacute phase, impaired mitochondrial respiration, and inadequate supply of acetyl-CoA during muscle fatigue.

## Graphical abstract

**Figure:**
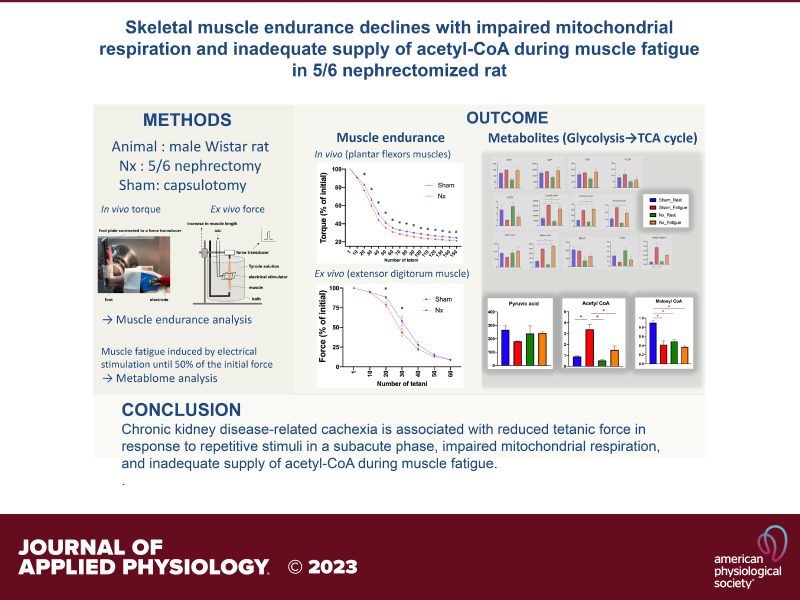

## INTRODUCTION

Chronic kidney disease (CKD) is characterized by progressive damage and dysfunction in the kidneys. It has been widely recognized that the presence of CKD is associated not only with increased risks of developing cardiovascular disease and all-cause mortality but also with cachexia accompanied by malnutrition and muscle wasting with muscle weakness and fatigue (1). It has been shown that muscle fatigue is more directly related than muscle weakness to accidental falls (2, 3). In previous studies on CKD-related reduction in skeletal muscle endurance, muscle performance was simply assessed by using a grip test and treadmill running (4, 5). Since whole body exercise is influenced by respiratory function, circulation, and nerve systems, exercise such as treadmill running has a limitation for assessing the physiological characteristics of muscle fatigue that occurs in skeletal muscle alone. Thus, although it has been widely recognized that decreased muscle endurance is associated with CKD-related cachexia, its physiological characteristics and underlying mechanisms remain unclear. We previously confirmed the importance of muscle physiological assessment using electrical stimulators to elucidate the pathophysiology of skeletal muscle in animal models of various diseases (6–8). The first aim of this study is to characterize the physiology of muscle endurance in CKD independently of the effects of whole body exercise.

Mitochondria play a central role in muscle metabolism and are closely related to physiological muscle endurance. Mitochondrial dysfunction in skeletal muscle has recently been highlighted as a major pathophysiological factor of CKD-associated muscle fatigue (4, 9, 10). In those previous studies, several mitochondrial dysfunctions including impairment of oxidative phosphorylation (OXPHOS) and decreased expression of key enzymes in the tricarboxylic acid (TCA) cycle were found. However, it remains unclear how these mitochondrial dysfunctions are associated with physiological characteristics of decreased skeletal muscle endurance. Furthermore, it is not known how the mitochondria-related metabolites change during muscle fatigue in CKD. Recently, metabolomic analysis before and after exercise using blood and urine samples has revealed notable changes during exercise in specific metabolites in various diseases (11–13). A few studies using healthy skeletal muscles have revealed differences in energy metabolism before and after exercise (14, 15). However, such studies also have the limitation that results of metabolome analysis in voluntary exercise such as running are highly dependent on the state of the subject’s respiratory and circulatory system at the time of sampling, leading to the possibility that the effect of exercise load cannot be strictly constant for each subject’s muscles (16).

In the present study, to clarify the physiological characteristics of muscle endurance and the metabolic responses during skeletal muscle fatigue in the condition of mitochondrial dysfunction caused by CKD, we assessed *1*) muscle endurance using both in vivo and ex vivo systems, *2*) muscle mass and fiber type-specific myofilament protein isoforms, *3*) functions and contents of mitochondrial OXPHOS, and *4*) metabolomic profiling between resting and fatigued state in hindlimb skeletal muscles from a 5/6 nephrectomized rat model of CKD. We hypothesized that CKD model rats have a reduced muscle endurance in association with mitochondrial dysfunction and that the identifying abnormal responses of mitochondria-related metabolites associated to muscle fatigue would allow us to clarify sites of impaired energy metabolism during muscle fatigue that should be targeted for intervention in CKD-related cachexia.

## METHODS

### Experimental Approval

All experimental protocols were reviewed and approved by the Ethics Committee on Animal Experiments of Sapporo Medical University (No. 20-076, Sapporo, Japan). Animal care was performed in strict accordance with institutional guidelines.

### Animals and Experimental Design

**Figure 1.**
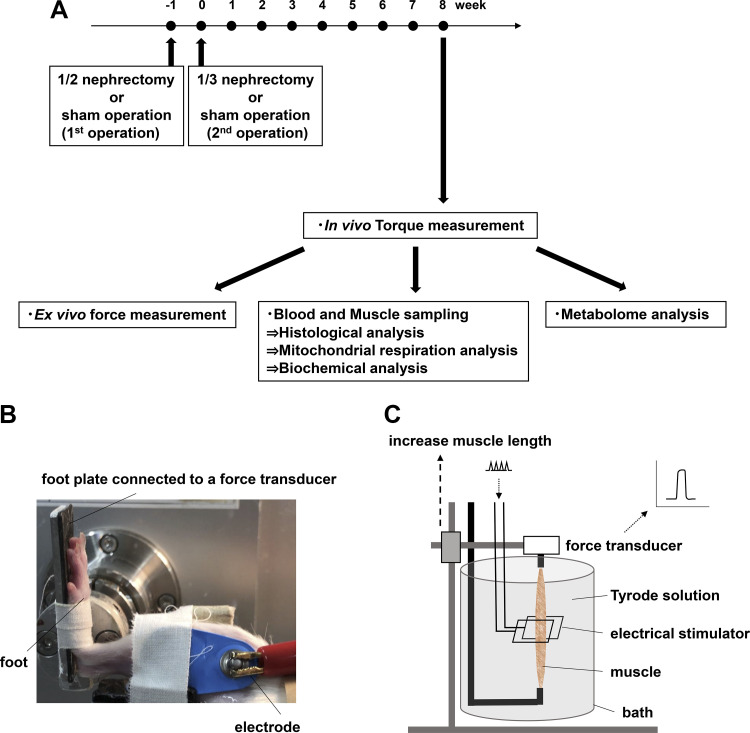
Experimental protocol and equipment for in vivo torque and ex vivo force measurements. Timeline of surgical procedures, in vivo torque measurements, and following examinations (*A*). Photograph of the setup for in vivo torque measurement (*B*). In vivo torque of isometric contractions produced in response to electrical stimulation with surface electrodes was measured. The left leg was attached to a foot plate connected to a torque sensor. Schematic image for ex vivo force measurement (*C*). Ex vivo force of isometric contractions produced by field electrical stimulation in a bath containing Tyrode solution was measured.

A total of 46 male Wistar rats (6 wk old) were supplied by Sankyo Labo Service (Sapporo, Japan) and acclimated for 7 days before being used in experiments. The rats were given food and water ad libitum and housed in an environmentally controlled room (24 ± 2°C) with a 12-h light-dark cycle. After acclimation, they were allocated to two different groups based on a randomly assigned serial number when they were brought in: a sham-operated control (Sham) group (*n* = 23) and a 5/6 nephrectomy (Nx) group (*n* = 23). The timeline of the surgical procedures and following examinations is shown in Fig. 1*A*. The operation for Nx was carried out by following a two-step procedure that reduces the original renal mass by five-sixths as previously described (17). Briefly, in the first step as 1/2 nephrectomy, the right kidney was removed via a right flank incision under anesthesia with 2% inhaled isoflurane. At 1 wk after the procedure, in the second step as 1/3 nephrectomy, 2/3 of the remaining kidney was removed by resecting the upper and lower poles of the left kidney via a left flank incision. Bleeding was controlled with an adhesive agent for tissue (Spongel, LTL Pharma, Japan). Sham rats only received capsulotomy with the same operation duration as that in Nx rats. Thereafter, detailed physiological assessments and biochemical analyses of skeletal muscle were performed, all using only the left legs of Sham rats and Nx rats. Eight weeks after the second operation, in vivo torque measurement was examined. Twenty-four hours after the measurement, the rats were randomly allocated to subsequent experiments including ex vivo force measurement, blood and muscle samplings for histological analysis, biochemical analysis, mitochondrial respiration analysis, and metabolome analysis. For blood and muscle samplings, rats were anesthetized with 2% inhaled isoflurane and cardiac puncture with a 20-gauge needle was performed to collect blood samples. Following the blood sampling, rats were securely euthanized by cervical dislocation. The plantar flexor muscles including gastrocnemius, plantaris (PLA), soleus muscles were collected and weighed, respectively. The PLA muscle was immediately excised and used for histological analyses or frozen in liquid nitrogen and stored at −80°C for biochemical analyses. For ex vivo force measurements, the extensor digitorum longus (EDL) muscle was adopted because PLA muscle is not technically suitable for accurate tension evaluation due to its pinnate-shaped nature. For metabolome analysis, apart from the collection of PLA muscle at rest, PLA muscle in fatigued state was collected immediately when the tension was reduced to 50% of the initial torque by fatigue stimulation using electrical stimulation, and the muscle was frozen in liquid nitrogen for the next procedures. Estimated glomerular filtration rate (eGFR) (µL/min) from blood sample data was calculated using the following equations (18): Plasma creatinine <52 µmol/L: eGFR = 880 × *W*^0.695^ × *C*^−0.660^ × *U*^−0.391^, Plasma creatinine ≥52 µmol/L: eGFR = 5862 × *W*^0.695^ × *C*^−1.150^ × *U*^−0.391^. *W* is weight (g), *C* is creatinine concentration (mmol/L), and *U* is urea (mmol/L).

### In Vivo Torque Measurement with Electrical Stimulation

#### Setting and torque measurement.

A photograph of the setup for in vivo torque measurement is shown in Fig. 1*B*. The torques were recorded on a computer and analyzed using LabChart version 8. Rats were anesthetized by 2% isoflurane inhalation and each rat was placed supine on a platform and the left foot was secured in a foot plate connected to a torque sensor (S-14154, Takei Scientific Instruments, Japan) at an angle of 0° plantarflexion. Plantar flexor muscles including the gastrocnemius, PLA, and soleus muscles were stimulated supramaximally (45 V) using a pair of surface electrodes (BlueSensor, Ambu, surface area of 0.785 cm^2^) that were placed on the skin and strapped by tape to the posterior surface of the calf. The maximum in situ torque was measured at a stimulation frequency of 100 Hz (duration 600 ms) with short (0.5 ms) current pulses such that only one action potential was triggered by each pulse as previously described (19). Specific torque was calculated as the ratio of in situ torque to the whole muscle weight for plantar flexors.

#### Measurement of in vivo muscle endurance.

In vivo muscle endurance was measured by 180 repeated 350-ms, 70-Hz tetani given at an interval of 2 s.

### Ex Vivo Force Measurement Using Electrical Stimulation

#### Setting of ex vivo force.

A freshly isolated EDL muscle was mounted between a force transducer (Nihon Kohden, Japan) and an adjustable holder and was filled with Tyrode’s solution (mM): NaCl, 121; KCl, 5; CaCl_2_, 1.8; MgCl_2_, 0.5; NaH_2_PO_4_, 0.4, NaHCO_3_, 24; EDTA, 0.1; glucose, 5.5. The solution was bubbled with 5% CO_2_-95% O_2_, which gives an extracellular pH of 7.4, and kept at 30°C. Supramaximal 1.0-ms monophasic rectangular pulses were applied via two platinum plate electrodes placed on both sides of the muscle. Muscle length was adjusted to the length (*L*_0_) giving maximum tetanic force and measured with a digital caliper. A schematic image for ex vivo force measurement is shown in Fig. 1*C*.

#### Measurement of ex vivo muscle endurance.

Ex vivo muscle endurance was measured by 60 repeated 350-ms, 70-Hz tetani given at an interval of 2 s.

### Histological Analysis

For histological analysis, the middle belly of the PLA muscle was frozen in precooled isopentane and stored at −80°C until the following steps. Cryostat sections (10 µm in thickness) were stained with hematoxylin and eosin (HE). HE images were captured from the serial sections using a fluorescence microscope (BIOREVO BZ-CE, KEYENCE, Japan). Digitally captured images (magnification ×20) with a minimum of three fields-of-view per muscle cross-section were processed and analyzed using ImageJ software (NIH, Bethesda, MD). The values were calculated from ∼30% of the total area by area, using 4–6 fields-of-view, counting more than 400 myofibers from randomly selected fields-of-view within frozen sections taken from the center of the muscle belly. We counted muscle fiber cross-sections and normalized these data to the number of muscle fibers, as previously reported (20).

### Biochemical Analysis

To obtain whole muscle protein lysates, pieces of the PLA muscle were homogenized in ice-cold homogenizing buffer (30 µL/mg wet wt) consisting of (in mM): Tris maleate, 10; NaF, 35; NaVO_4_, 1; 1% Triton X 100 (vol/vol), and 1 tablet of protease inhibitor cocktail (Roche) per 50 mL. Muscle protein lysates were stored at −80°C until the following biochemical experiments.

#### Separation of myosin heavy chain isoforms.

Aliquots of the homogenized muscles were used for myosin heavy chain (MHC) electrophoresis as previously described in detail (21). Using a 6% polyacrylamide slab gel, electrophoresis was run at 4°C for 24 h at 160 V and staining with Coomassie brilliant blue was performed. Images of gels were densitometrically quantified with ImageJ.

#### Western blots.

The homogenized aliquot was centrifuged at 14,000 *g* for 15 min at 4°C to obtain the supernatant. The protein content was determined using a bicinchoninic acid (BCA) Protein Quantification Kit (Takara-bio, Japan). Equal amounts of protein were resolved and loaded using NuPAGE Novex 4–12% Bis-Tris midi gels (Thermo Fisher Scientific, Waltham, MA) and transferred to nitrocellulose membranes. Total proteins on the membrane were visualized by 0.1% (wt/vol) of Ponceau S in 5% acetic acid. After blocking with Tris-buffered saline (TBS) containing 0.005% Tween 20 and 5% milk, the membranes were incubated overnight with primary antibodies against oxidative phosphorylation (OXPHOS) antibody cocktail (Abcam 110413, RRID:AB_2629281, 1:250 dilution), NDUFS1 (Santa Cruz 271387, RRID:AB_10611343, 1:1,000 dilution), Voltage-dependent anion-selective channel 1 (VDAC1) (Proteintech 5529-1-AP, RRID:AB_10837225, 1:1,000 dilution), cytochrome c oxidase (COX) IV (Abcam 16056, RRID:AB_443304, 1:1,000 dilution), and prohibitin (Abcam 28172, RRID:AB_777457, 1:500 dilution). Horseradish peroxidase-conjugated anti-mouse (1:4,000 dilution) or anti-rabbit (1:5,000 dilution) secondary antibodies were purchased from Bio-Rad (Hercules, CA). The blots were developed using the Pierce ECL reagent (Thermo Fisher Scientific, Waltham, MA). Images were photographed and processed using a ChemiDoc XRS+System with Image Lab software (Bio-Rad, Hercules, CA). Intensities of individual bands were normalized by total proteins detected by Ponceau S staining and quantified by using ImageJ software.

### Measurements of Mitochondrial Respiration

Oxygen consumption rates of freshly isolated mitochondria from the PLA muscle were measured using Seahorse XFe96 Bioanalyzer as previously described with slight modifications (22). In brief, muscles that had been quickly excised from rats were cut into small pieces in ice-cold fiber relaxation buffer (100 mM KCl; 5 mM EGTA; 5 mM HEPES, pH adjusted to 7.0 with KOH). The muscle pieces were then homogenized in HES buffer (5 mM HEPES; 1 mM EDTA; 250 mM sucrose, pH adjusted to 7.4 with KOH) using a Dounce homogenizer. The homogenate was centrifuged for 10 min at 500 *g* twice and the supernatant was centrifuged at 9,000 *g* for 15 min at 4°C. The pellet including crude mitochondria was re-suspended in MAS buffer (70 mM sucrose; 220 mM mannitol; 10 mM KH_2_PO_4_; 5 mM MgCl_2_; 2 mM HEPES; 1 mM EGTA; 0.2% fatty acid-free BSA, pH adjusted to 7.4 with KOH). The protein concentration was measured using a BCA Protein Quantification Kit (Takara-bio, Japan). Since the buffer contains BSA, the mitochondrial protein concentration was determined by subtracting background BSA protein content. Equal amounts of mitochondria in MAS buffer (10 µg) were loaded in each well for pyruvate/malate-driven respiration and succinate-driven respiration. The plates were centrifuged at 1,400 *g* for 20 min. Following centrifugation, MAS buffer containing 5 mM pyruvate and 5 mM malate or 5 mM sodium succinate and 2 µM rotenone was added to a final volume of 180 µL and incubated for 8 min at 37°C without CO_2_. Oxygen tension was measured in the Seahorse XFe96 Bioanalyzer at baseline and following injections of 20 mM ADP, 10 μM oligomycin, and 5 μM rotenone/antimycin.

### Metabolome Analysis

#### Sampling muscles in resting and fatigued states.

Sham rats and Nx rats for muscle sampling in resting state were anesthetized with 2% inhaled isoflurane and securely euthanized by cervical dislocation. The PLA muscle was immediately excised from the left leg of the rats and frozen in liquid nitrogen and stored at −80°C. On the other hand, to collect muscles in a fatigued state, the left legs of Sham rats and Nx rats were stimulated by repeated 70 Hz tetani of 350 ms in duration at 2-s intervals using the in vivo torque measurement system as described earlier. The stimulation was stopped when muscle tension reached 50% of the initial force, the rat was quickly euthanized, and the PLA muscle was immediately isolated. The isolated PLA muscle was then immediately frozen in liquid nitrogen and stored at −80°C until the day of the experiment.

#### Extraction of metabolites.

Extraction and measurement of metabolites excluding glycogen assay were performed in the faculty of Human Metabolome Technologies (HMT, Tsuruoka, Yamagata, Japan) Inc. Approximately 20–40 mg of frozen tissue was placed in a homogenization tube together with zirconia beads (5 mmφ and 3 mmφ). Next, 1,500 µL of 50% acetonitrile/Milli-Q water containing internal standards (H3304-1002, HMT) was added to the tube, after which the tissue was completely homogenized at 1,500 rpm for 60 s at 4°C using a beads shaker (Shake Master NEO, Bio Medical Science, Tokyo, Japan). The homogenate was then centrifuged at 2,300 *g* for 5 min at 4°C. Subsequently, 800 µL of the upper aqueous layer was centrifugally filtered through a Millipore 5-kDa cutoff filter (UltrafreeMC-PLHCC, HMT) at 9,100 *g* for 180 min at 4°C to remove macromolecules. The filtrate was evaporated to dryness under vacuum and reconstituted in 50 µL of Milli-Q water for metabolome analysis at HMT.

#### Measurements of metabolites.

Concentrations of extracted metabolites were measured by capillary electrophoresis time-of-flight mass spectrometry (CE-TOFMS) and capillary electrophoresis tandem mass spectrometry as described previously (23). Metabolome analysis was conducted according to HMT’s C-SCOPE package, using CE-TOFMS for cation analysis and CE-tandem mass spectrometry (CE-MS/MS) for anion analysis based on methods described previously (24). Briefly, CE-TOFMS and CE-MS/MS analyses were carried out using an Agilent CE capillary electrophoresis system equipped with an Agilent 6210 time-of-flight mass spectrometer (Agilent Technologies, Inc., Santa Clara, CA) and Agilent 6460 Triple Quadrupole LC/MS (Agilent Technologies), respectively. The systems were controlled by Agilent G2201AA ChemStation software version B.03.01 for CE (Agilent Technologies) and connected by a fused silica capillary (50 μm in i.d. × 80 cm in total length) with commercial electrophoresis buffers (H3301-1001 and I3302-1023 for cation and anion analyses, respectively, HMT) as the electrolytes. The time-of-flight mass spectrometer was scanned from *m*/*z* 50 to 1,000 and the triple quadrupole mass spectrometer was used to detect compounds in dynamic MRM mode. Peaks were extracted using MasterHands automatic integration software (Keio University, Tsuruoka, Yamagata, Japan) (25) and MassHunter Quantitative Analysis B.04.00 (Agilent Technologies) to obtain peak information including *m*/*z*, peak area, and migration time (MT). Signal peaks were annotated according to HMT’s metabolite database based on their *m*/*z* values and MTs. The peak area of each metabolite was normalized to internal standards, and the metabolite concentration was evaluated by standard curves with three-point calibrations using each standard compound. Principal component analysis (PCA) and hierarchical cluster analysis (HCA) (26) were performed by HMT’s proprietary R programs and MATLAB, respectively.

#### Glycogen assay.

Glycogen concentrations of muscles in resting and fatigued states were measured using fluorometric techniques as previously described (27). Approximately 30 mg of frozen muscle tissue was placed in a homogenization tube and the sample was extracted in 0.5 mL of 2 N HCl at 100°C for 2 h and then neutralized with 0.5 mL of 2 N NaOH. The assay mixture was composed of 1 M Tris-HCl, pH 8.1, 0.1 mM MgCl_2_, 0.5 M DTT, 300 mM ATP, 50 mM NADP, 0.07 U/mL glucose-6-phosphate dehydrogenase, and 0.17 U/mL hexokinase. Glycogen content was normalized to muscle wet weight.

### Enzyme Activity Assay for PDH

Mitochondrial extraction was carried out as previously described (22) from rats skeletal muscle isolated using Mitochondria Isolation Kit (Pierce, Rockford, IL). The protein concentration was determined using a BCA Protein Quantification Kit (Takara-bio, Japan). Mitochondria protein was used for pyruvate dehydrogenase (PDH) activity assay according to the supplier’s manual. Mitochondrial protein (0.8 µg) was used for PDH assay (Catalogue Number MAK183, Sigma-Aldrich). Assay solutions were added to the plate before reading the absorbance of each well at 450 nm using a kinetic program with 20 s between reads (plate reader).

### Statistical Analyses

All results are presented as mean with SEM or mean with 95% confidence interval. Results were analyzed by Welch’s *t* test for experiments comparing two groups. When more than two groups were compared, one-way ANOVA followed by Tukey’s test or two-way ANOVA followed by Sidak’s test was used to analyze differences between groups. Kolmogorov–Smirnov test was used for comparison of frequency distributions about muscle fiber area. All statistics were performed on GraphPad Prism 8 and differences were considered statistically significant at *P* < 0.05 (*). A power analysis was performed using SigmaPlot (v.13, Systat Software, Inc.), assuming changes in physiological measurements for Nx rats being 30 ± 20% of the value for Sham rats. With a power of 0.80 and an α of 0.05, this gives a sample size of five. Based on this, we used 5 and 6 animals in each group.

## RESULTS

### Muscle Endurance, as Assessed by In Vivo and Ex Vivo Measurements, is Reduced in CKD

First, we assessed physical and biochemical parameters in Sham and Nx rats. As shown in Table 1, compared with Sham rats, Nx rats had significantly decreased body weight (Sham: 340.3 ± 3.6 vs. Nx: 276.2 ± 5.6 g) and wet weight of plantar flexor muscles (Sham: 2,092 ± 51 vs. Nx: 1,650 ± 201 mg). Nx rats also drank significantly more water than Sham rats (Sham: 26.2 ± 0.4 vs. Nx: 51.9 ± 1.4 g/24 h), but the food intake did not show significant difference between Nx rats and Sham rats (Sham: 16.5 ± 0.4 vs. Nx: 14.4 ± 0.8 g/24 h). Serum tests showed that Nx rats had significantly higher levels of blood urea nitrogen (4.1-fold) and serum creatinine (4.5-fold), and significantly lower levels of eGFR (0.09-fold) and hemoglobin (0.9-fold). These findings indicate that the Nx rats used in the present study mimic the clinical phenotype of patients with CKD.

**Table 1.** Biometrical and biochemical parameters in Sham and Nx rats

	Body Weight, g	Plantar Flexors, mg	Food Intake, g/24 h	Water Intake, g/24 h	BUN, mg/dL	s-Cre, mg/dL	eGFR, µL/min	Hemoglobin, g/dL
Sham	340.3 ± 3.6	2092 ± 51	16.5 ± 0.4	26.2 ± 0.7	21.5 ± 0.4	0.29 ± 0.01	2678 ± 92	14.0 ± 0.3
Nx	276.2 ± 5.6*	1650 ± 201*	14.4 ± 0.8	51.9 ± 1.4*	109.6 ± 6.1*	1.61 ± 0.13*	243 ± 30*	12.3 ± 1.0*

Data are expressed as means ± SE in Sham rats (*n* = 6) and 5/6 nephrectomy (Nx) rats (*n* = 6). Plantar flexors mean the sum of gastrocnemius, plantaris, and soleus muscle weights. BUN, blood urea nitrogen; eGFR, estimated glomerular filtration rate; s-Cre, serum-creatinine. *P < 0.05 with Welch’s *t* test for comparison of two groups, Sham vs. Nx.

**Figure 2.**
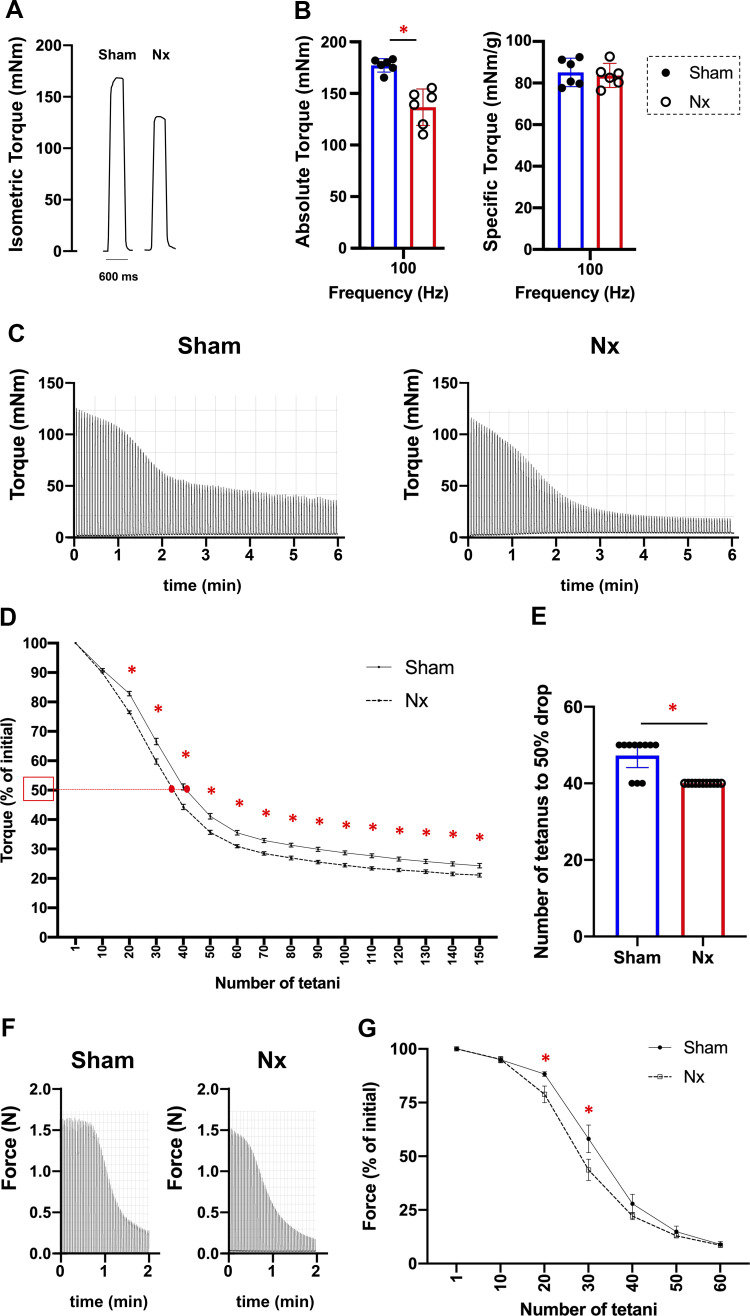
Characteristics of muscle tension and muscle fatigue in chronic kidney disease (CKD). Representative original records 100-Hz tetanic torque in plantar flexor muscles in vivo from Sham rats and 5/6 nephrectomy (Nx) rats (*A*). Absolute torque in plantar flexor muscles and specific torque (determined as the ratio of absolute torque to the weight of whole plantar flexor muscle) at 100-Hz stimulation frequency in Sham rats (*n* = 6) and Nx rats (*n* = 6) (*B*). Representative torque records during the fatigue protocol (180 repeated 70-Hz, 350-ms tetani at 2-s intervals) of the plantar flexor muscles in vivo from Sham rats and Nx rats (*C*). In vivo fatigue development of plantar flexor muscles in Sham rats (*n* = 11) and Nx rats (*n* = 11) (*D*). Torque in the first tetanus was set to 100% in each muscle. Red filled dots mean the points at 50% of their initial torque for each group. At these points, the stimulations were stopped and the plantaris muscles in fatigued state were sampled for metabolome analysis. The number of tetanic stimulations needed until the force was decreased to 50% of the initial torque in the in vivo fatigue stimulations (*E*). Representative force records during the fatigue protocol (60 repeated 70-Hz, 350-ms tetani at 2-s intervals) of the extensor digitorum longus (EDL) muscle ex vivo from Sham rats and Nx rats (*F*). Ex vivo fatigue development of EDL muscles in Sham rats (*n* = 5) and Nx rats (*n* = 6) (*G*). *P < 0.05 with Welch’s *t* test for comparison of two groups (*B* and *E*) and two-way repeated-measures ANOVA with Sidak’s post hoc test for comparison of two groups during muscle endurance (*D* and *G*), Sham vs. Nx. Data are shown as mean and SEM or mean and 95% confidence interval with individual values.

Next, in situ torque and muscle fatigue in the plantar flexor muscles were assessed using an in vivo torque measurement apparatus. Figure 2*A* shows representative isometric torques of the plantar flexor muscles of Sham and Nx rats stimulated at a high frequency of 100 Hz. The absolute torque was significantly decreased in Nx rats compared with that in Sham rats [136.6 (118.1–155.1) vs. 177.1 (170.2–183.9) mN·m], but the specific torque that was normalized by the weight of the whole plantar flexor muscles did not show the significant difference [Nx: 83.7 (77.9–89.4) vs. Sham: 85.1 (78.3–92.0) mN·m/g] (Fig. 2*B*). Figure 2*C* shows representative records of torque induced by in situ repeated tetanic stimulation of the plantar flexor muscles of Sham and Nx rats. In the muscle fatigue examination using the in situ intermittent electrical stimulation, it was showed a significant interaction between the groups and the number of tetanic stimulation (*P* < 0.0001), with greater rate of fatigue development in Nx rats in response to 20 or more repetitive stimuli (Fig. 2*D*). The number of repetitive stimuli until the force was decreased to 50% of the starting force was also significantly smaller in Nx rats than in Sham rats [40.0 (40.0–40.0) vs. 47.3 (44.1–50.4) times] (Fig. 2*E*). Furthermore, to examine whether muscle fatigue occurred independently of hemodynamics, electrolytes, uremic substances, hemoglobin levels, and intra-blood metabolites in CKD, the endurance of the EDL muscle as a representative muscle of fast-twitch muscles was evaluated using ex vivo force measurement system. Figure 2*F* shows representative torque records obtained during fatigue induced by ex vivo repeated tetanic stimulation of the EDL muscles from Sham rats and Nx rats. In the EDL muscles of Nx rats, it was shown a significant interaction between the groups and the number of tetanic stimulation (*P* < 0.0004), with greater rate of fatigue development in EDL muscles of Nx rats in response to 20 and 30 repetitive stimuli compared with that in EDL muscles of Sham rats (*P* < 0.0224) (Fig. 2*G*). These findings indicate that skeletal muscles in CKD have decreased muscle endurance in response to 20 or more repetitive electrical stimuli in both in vivo and ex vivo conditions.

### Muscle Mass is Reduced without Myofiber-Type Transition in CKD

**Figure 3.**
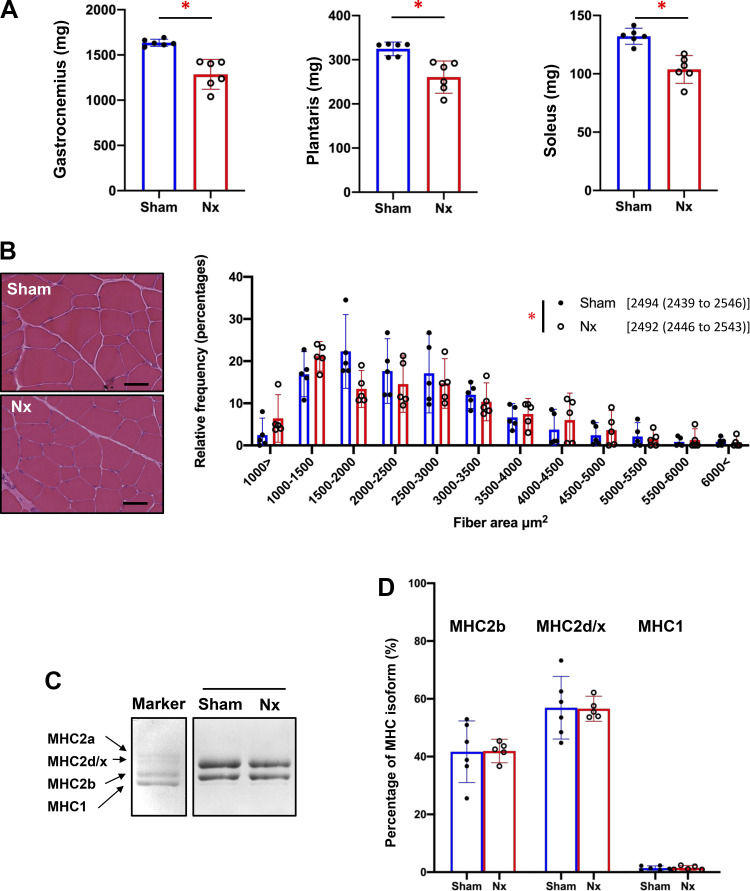
Muscle wasting without a transition of myofiber type in chronic kidney disease (CKD). *A* and *B:* muscle mass of plantar flexor muscles and fiber area in a fast-twitch plantaris (PLA) muscles, respectively. The muscle wet weight of plantar flexor muscles including gastrocnemius, PLA, soleus muscles from Sham rats (*n* = 6) and 5/6 nephrectomy (Nx) rats (*n* = 6) (*A*). Representative cross-sectional image stained with hematoxylin and eosin, and frequency histogram showing the distribution of muscle fiber area in PLA muscles of Sham rats (*n* = 6) and Nx rats (*n* = 6) (*B*). Black scale bars in the images indicate 50 μm. The mean, SEM, and statistical comparison are shown in the histogram legend. *C* and *D:* muscular fiber-type distributions. Representative images of electrophoretic separation of myosin heavy chain (MHC) isoforms of the PLA muscle (*C*). In the marker lane, a mixture of a fast-twitch extensor digitorum longus muscle and a slow-twitch soleus muscle isolated from rats was applied as an indicator of each isoform. Proportions of MHC isoforms in Sham rats (*n* = 6) and Nx rats (*n* = 5) are shown (*D*). *P < 0.05 with Welch’s *t* test for comparison of two groups (*A* and *D*) and Kolmogorov–Smirnov test for comparison of frequency distributions of two groups (*B*), Sham vs. Nx. Data are shown as mean and 95% confidence interval with individual values.

Next, we assessed whether decreased muscle endurance observed in Nx rats was accompanied by changes in muscle mass or myofiber type transition. As shown in Fig. 3*A*, Nx rats showed significant reductions in wet weight of the plantar flexor muscles compared with Sham rats [gastrocnemius muscle: 1,286 (1,121–1,451) vs. 1,635 (1,596–1,674) mg; PLA muscle: 261 (224–297) vs. 325 (309–340) mg; soleus muscle: 104 (92–116) vs. 132 (125–139) mg]. In HE staining of the PLA muscles, frequency distribution analysis indicated that Nx rats had significantly more small diameter muscle fibers than Sham rats [2,492 (2,446–2,543) vs. 2,494 (2,439–2546) µm^2^, *P* < 0.0001] (Fig. 3*B*). Moreover, homogenates of PLA muscles were subjected to gel electrophoresis to assess myofiber type transition. The proportions of fiber type-specific MHC isoforms (MHC1: slow-type, MHC2a: intermediate-type, MHC2d/x: fast-type, MHC2b: fast-type) were comparable in Sham rats and Nx rats [MH1: 1.41 (0.65–2.17)% vs. 1.48 (0.64–2.32)%, *P* = 0.8756; MH2d/x: 56.92 (46.07–67.77)% vs. 56.57 (52.22 to 60.92)%, *P* = 0.9439; MH2b: 41.67 (30.99–52.35)% vs. 41.95 (37.89–46.02)%, *P* = 0.9538] (Fig. 3, *C* and *D*). These results indicate that CKD can induce muscle atrophy without myofiber-type transition.

### Mitochondrial Respiratory Capacity is Impaired in CKD along with Decreased Expression of Electron Transport Chain Subunits

**Figure 4.**
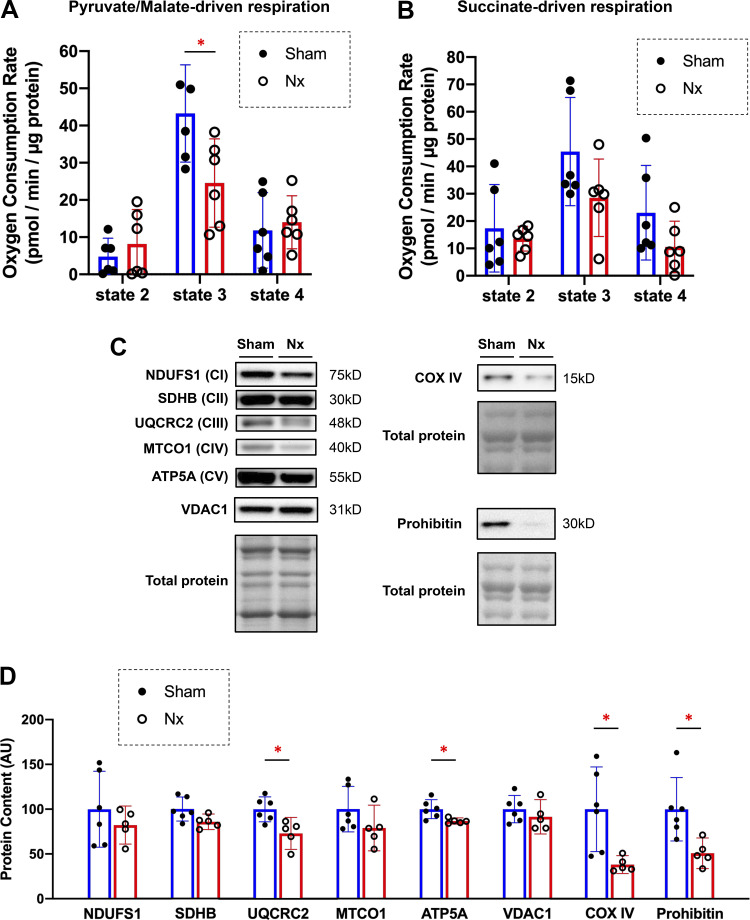
Capacity and contents of mitochondrial oxidative phosphorylation in chronic kidney disease (CKD). *A* and *B:* oxygen consumption rates determined by the Seahorse XFe96 analyzer in mitochondria isolated from fast-twitch plantaris (PLA) muscles in Sham and 5/6 nephrectomy (Nx) rats. Pyruvate/malate-driven respiration in the PLA muscle (*A*) *n* = 6 in each. Succinate-driven respiration in the PLA muscle (*B*) *n* = 6 in each. State 2 respiration indicates baseline respiration, state 3 respiration indicates oxygen consumption with ADP, and state 4 respiration indicates respiration with oligomycin. Representative Western blots (*C*) and their densitometries (*D*) showing the contents of oxidative phosphorylation complex proteins (complexes I–IV), Voltage-dependent anion-selective channel 1 (VDAC1), cytochrome c oxidase (COX) IV, and prohibitin in the PLA muscles in Sham rats (*n* = 6) and Nx rats (*n* = 5). *P < 0.05 with Welch’s *t* test for comparison of two groups, Sham vs. Nx. Data are shown as mean and 95% confidence interval with individual values. Full-length blots/gels are presented in Supplemental Fig S2.

To assess the involvement of mitochondrial respiratory capacity in muscle fatigue in CKD, the oxygen consumption rate in freshly isolated mitochondria from the PLA muscle was evaluated using an extracellular flux analyzer. State 3 respiration (ADP-stimulated respiration) using pyruvate/malate as substrates was significantly lower in Nx rats than in Sham rats [43.3 (30.2–56.3) vs. 24.6 (12.7–36.4) pmol/min/µg protein], whereas the respiration using succinate as a substrate showed a lowering trend but did not reach significance in Nx rats than in Sham rats [45.4 (25.7–65.2) vs. 28.5 (14.4–42.7) pmol/min/µg protein]. State 2 respiration (basal respiration) [pyruvate/malate: 4.8 (−0.2 to 17.4) vs. 8.2 (−1.0 to 17.4) pmol/min/µg protein; succinate: 17.4 (1.4–33.4) vs. 13.6 (9.0–18.1) pmol/min/µg protein] and state 4 respiration (leak respiration) [pyruvate/malate: 11.8 (1.7–22.0) vs. 14.0 (6.9–21.1) pmol/min/µg protein; succinate: 23.0 (5.7–40.3) vs. 10.5 (1.1–20.0) pmol/min/µg protein] were comparable in Sham rats and Nx rats (Fig. 4, *A* and *B*). Moreover, the expression levels of mitochondrial electron transfer chain complexes were assessed by Western blots. Expression levels of UQCRC2 (complex III) and ATP5A (complex V) in the PLA muscle were significantly decreased by 27.0% and by 13.3% in Nx rats compared with those in Sham rats. respectively. Evaluation of the expression levels of VDAC1, COX IV, and prohibitin, which reflect mitochondrial content, showed that COX IV and prohibitin in the PLA muscle were decreased by 61.7% and by 49.2% in Nx rats compared with those in Sham (Fig. 4*D*), although the difference of VDAC1 did not reach the statistical significance. These results suggest that mitochondrial respiratory capacity is impaired in the skeletal muscle accompanied by a decrease in some electron transfer chain complexes and mitochondria itself in CKD.

### Supply of Acetyl-CoA in Response to Muscle Fatigue is Impaired in CKD

**Figure 5.**
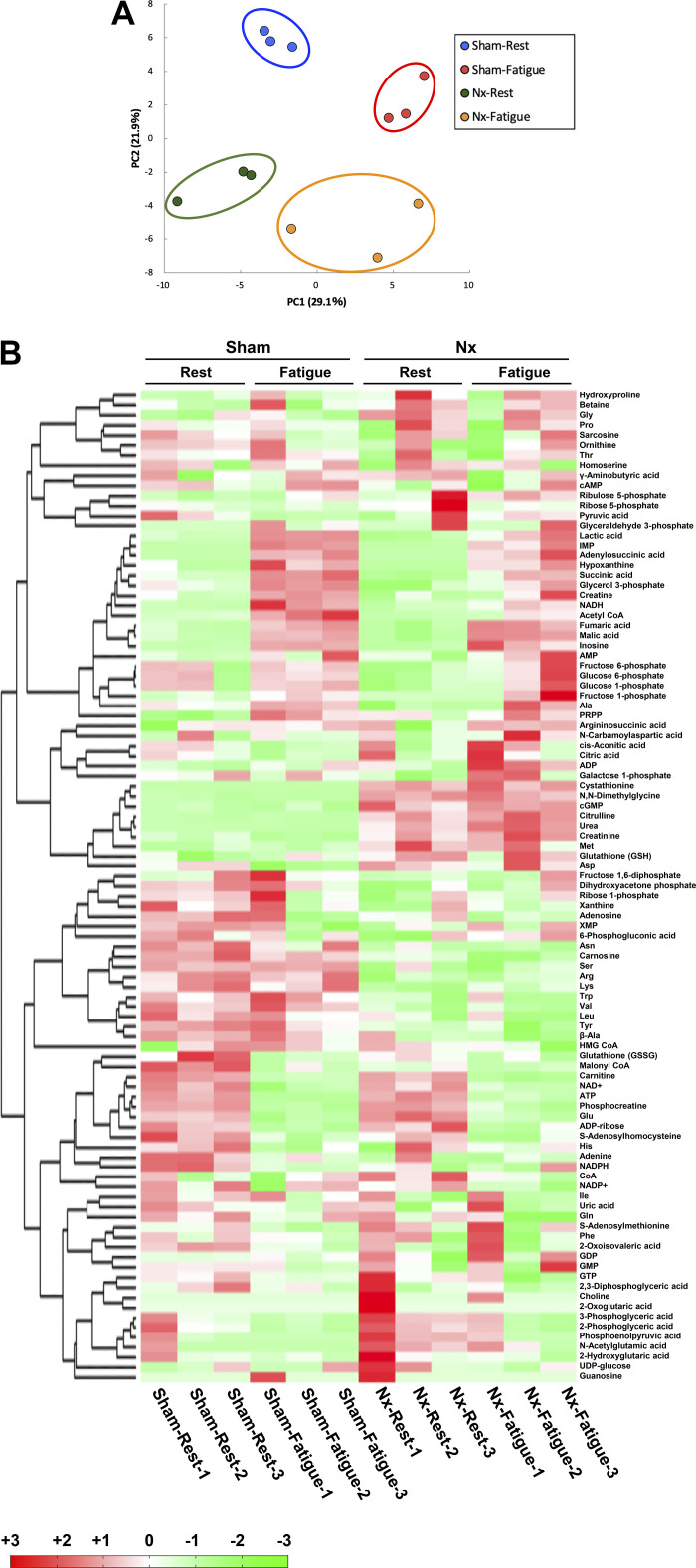
Characteristics of metabolites at rest and in a fatigued state in chronic kidney disease (CKD). Score plot of principal component analysis of the results of metabolome analysis in fast-twitch plantaris (PLA) muscles at rest and in a fatigued state in Sham and 5/6 nephrectomy (Nx) rats (*n* = 3 in each state) (*A*). Hierarchical clustering using the metabolites detected in the metabolome analysis is shown in a heatmap (*B*). The horizontal axis indicates each sample and the vertical axis indicates the peak reflecting the amounts of individual metabolites. Hierarchical clustering analysis was performed for the peaks, and the distance between the peaks is shown in the tree diagram on the left side of the heatmap. Values larger than the average are represented by darker red and values smaller than the average are represented by darker green.

Metabolome analysis was performed to identify metabolites related to mitochondria that were particularly impaired in response to fatigue in the PLA muscles of Sham and Nx rats. To obtain muscle samples in the fatigued state, electrical stimulation was applied to the in situ plantar flexor muscles until they were at 50% of their initial torque and the PLA muscle was isolated immediately after euthanasia (Fig. 2*D*). Of 116 metabolites that are measurable by CE-TOFMS and CE-MS/MS, 99 metabolites were detected in the muscle samples. The results of PCA clearly distinguished the resting and fatigued states in the first component, which accounted for 29.1% of the total variation (Fig. 5*A*). The second component, which accounted for 21.9% of the total variation, distinguished Sham and Nx rats (Fig. 5*A*). Changes in metabolites caused by CKD or fatigue induction were also visualized by hierarchical cluster analysis as a heatmap (Fig. 5*B*). The findings suggest that the presence of CKD or a fatigue state induces significant changes in the overall metabolite profile.

**Figure 6.**
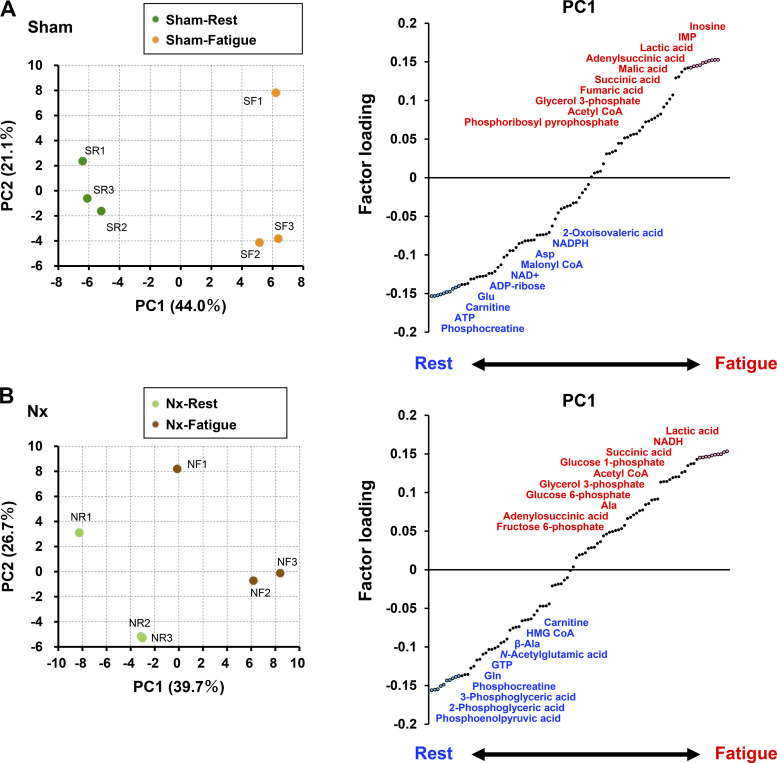
Principal component analysis between resting and fatigued states to reveal metabolic pathways that are impaired in chronic kidney disease (CKD). Score plots and factor loading plots of principal component analysis of the results of metabolome analysis between resting and fatigued states in a fast-twitch plantaris (PLA) muscles of Sham rats (*A*, *n* = 3 in each state; SR: Sham-Rest, SF: Sham-Fatigue) and in the PLA muscles of 5/6 nephrectomy (Nx) rats (*B*, *n* = 3 in each state; NR: Nx-Rest, NF: Nx-Fatigue). The upper right ten metabolites, colored red, indicate major increased substances post fatigue and the lower left ten metabolites, colored blue, indicate major decreased substances post fatigue in each right panel.

To assess more precisely the changes of intramuscular metabolites in response to fatigue in CKD, we compared metabolites at resting and fatigued state by PCA separately in Sham rats and Nx rats. In PCA, the first component, which accounted for 44.0% of the total variation in Sham rats (Fig. 6*A*) and 39.7% of the total variation in Nx rats (Fig. 6*B*), clearly separated the resting state from the fatigued state. The major segregating factors in the rest state has lower factor loading and greater metabolite decrease after fatigue, whereas the major segregating factors in the fatigued state has higher factor loading and greater metabolite increase after fatigue. In Sham rats, the major metabolites of which amounts changed in response to fatigue included end products of anaerobic glycolysis such as inosine, inosine monophosphate (IMP), and ATP and intermediates of the TCA cycle such as fumaric acid and malic acid (Fig. 6*A*). In Nx rats, the major metabolites of which amounts changed in response to fatigue included many intermediate products of glycolysis such as glucose 6-phosphate, fructose 6-phosphate, 3-phosphoglyceric acid, 2-phosphoglyceric acid, and phosphoenolpyruvic acid (Fig. 6*B*). The major metabolites in common in Sham and Nx rats that decreased in response to fatigue were phosphocreatine and carnitine, whereas the major metabolites that increased in response to fatigue were acetyl-CoA, glycerol 3-phosphate, succinic acid, adenylosuccinic acid, and lactic acid. These findings suggest that when CKD muscle becomes fatigued, there is a disturbance in the metabolic flux from the glycolytic system to the TCA cycle, in which acetyl-CoA is centrally located.

**Figure 7.**
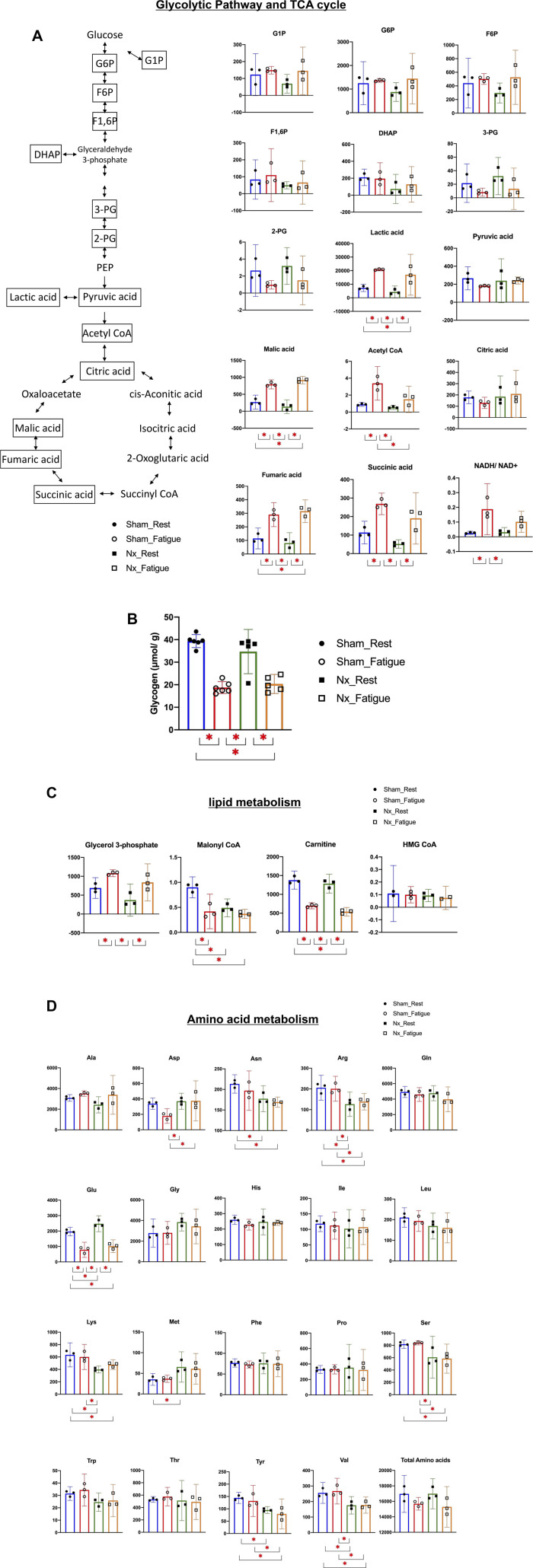
Individual metabolites related to the glycolytic pathway and TCA cycle at rest and in a fatigued state revealing deficiency in the supply of acetyl-CoA in chronic kidney disease (CKD). Individual metabolites of the glycolytic pathway and TCA cycles in a fast-twitch plantaris (PLA) muscle at rest and in a fatigue state in Sham and 5/6 nephrectomy (Nx) rats are shown on each side and schematic metabolic pathways are shown on the left side (*A*). Concentrations of metabolites are represented as nmol/g wet tissue. Glycogen contents assessed by a colorimetric glycogen assay (*B*). Individual metabolites related to lipid (*C*) and amino acids (*D*) in the PLA muscle at rest and in a fatigue state in Sham and Nx rats are shown. Concentrations of metabolites are presented as nmol/g wet tissue. DHAP, dihydroxyacetone phosphate; F1,6P, fructose 1,6-diphosphate; F6P, fructose 6-phosphate; G1P, glucose 1-phosphate; G6P, glucose 6-phosphate; PEP, phospohoenolpyruvic acid; 2-PG, 2-phosphoglyceric acid; 3-PG, 3-phosphoglyceric acid. *P < 0.05 with one-way ANOVA followed by Tukey’s post hoc test for comparison of groups. Data are shown as mean and 95% confidence interval with individual values.

To further elucidate which part of the metabolic flux is inhibited in CKD, individual metabolites of the major energy production pathways at rest and in a fatigued state were also evaluated. In the glycolytic pathway and TCA cycle, metabolites with statistically significant differences in amounts from resting to fatigued state were lactic acid (2.5-fold), malic acid (3.3-fold), acetyl-CoA (3.3-fold), fumaric acid (2.5-fold), succinic acid (2.5-fold), and NADH/NAD^+^ (2.5-fold) in Sham rats, whereas the changes in acetyl-CoA (2.5-fold) and NADH/NAD^+^ (3.3-fold) were impaired in Nx rats (Fig. 7*A*). Although the differences did not reach statistical significance, fatigue-induced decreasing trends in pyruvic acid and citric acid from resting to fatigued state observed in Sham rats (pyruvic acid: 0.7-fold; citric acid: 0.7-fold) were not observed in Nx rats (pyruvic acid: 1.0-fold: citric acid: 1.1-fold). Glycogen contents of PLA muscles in Sham rats and Nx rats were significantly decreased by 52.4% and 41.4% in response to fatigue, respectively (Fig. 7*B*). In lipid metabolism, metabolites with statistically significant differences in amounts from resting to fatigued state were glycerol 3-phosphate (1.6-fold), malonyl CoA (4.7-fold), and carnitine (0.5-fold) in Sham rats, whereas the change in malonyl CoA (0.8-fold) was impaired in Nx rats (Fig. 7*C*). In amino acid metabolism, the only metabolite with a statistically significant difference in the amount from resting to fatigued state was glutamic acid, and the magnitude of change was not different between Sham rats (0.4-fold) and Nx rats (0.4-fold) (Fig. 7*D*).

**Figure 8.**
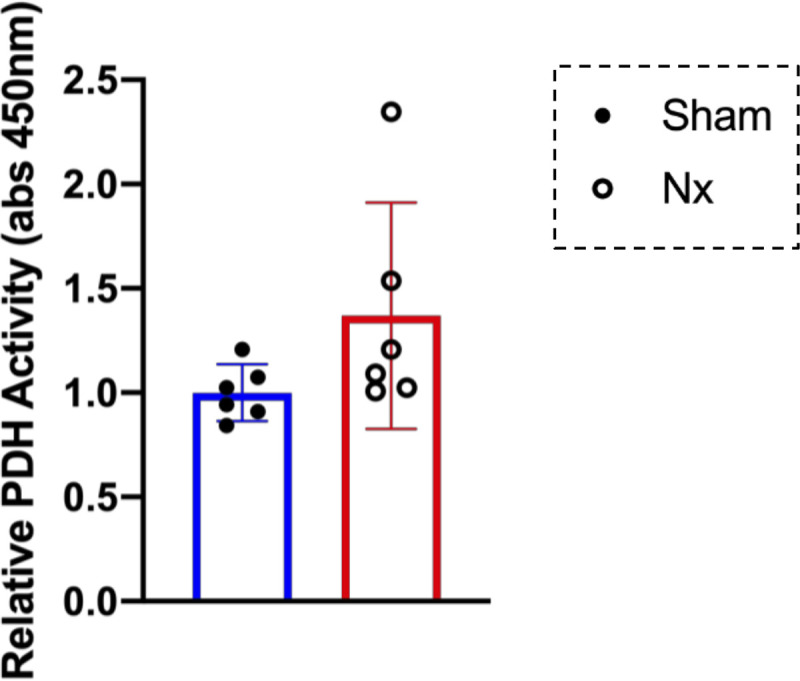
Pyruvate dehydrogenase activity in mitochondria in chronic kidney disease (CKD). Pyruvate dehydrogenase (PDH) activity in the mitochondria extracted from plantaris (PLA) muscles in Sham rats (*n* = 6) and 5/6 nephrectomy (Nx) rats (*n* = 6) measured by colorimetry *P < 0.05 with Welch’s *t* test for comparison of two groups, Sham vs. Nx. Data are shown as mean and 95% confidence interval with individual values.

There are numerous enzymes that can explain the differences in metabolic response to muscle fatigue in Sham and Nx. Among them, PDH is an essential enzyme for the production of acetyl-CoA, which is consumed in the TCA cycle supplying NADH and FADH2 to the electron transfer system, but its enzyme activity was not significantly different between Sham rats and Nx rats (*P* = 0.1442) (Fig. 8).

## DISCUSSION

CKD-related cachexia is closely associated with metabolic disturbances, causing decreased endurance and leading to functional loss of daily activities (28, 29). However, it has remained unclear how CKD affects the physiology of muscle fatigue and changes in mitochondrial function and intramuscular metabolites at rest and those in response to fatigue exercise. In the present study using a 5/6 nephrectomized CKD rat model, we found that CKD reduces muscle endurance without changes in the proportions of fiber type-specific MHC isoforms. Our results also showed that CKD induces mitochondrial OXPHOS dysfunction and inadequate supply of acetyl-CoA during muscle fatigue. These novel insights into the pathogenesis of CKD-related cachexia may assist in the development of effective interventions. Previous studies have shown that intramitochondrial accumulation of uremic toxins (10), production of reactive oxygen species (9), and mitochondrial DNA damage due to inflammation (30) caused by renal failure are key factors for decreased exercise performance. This study did not explore the upstream mechanisms of why renal failure caused the reduced muscle endurance and mitochondrial dysfunction. However, it does present valuable results that add to our understanding of metabolic disturbances, particularly those related to muscle fatigue. Abnormalities in these metabolic systems are also a topic of interest as an effect of urinary toxin accumulation (9, 10).

The findings obtained by muscle physiological experiments using in situ electrical stimulation clearly indicated decreased muscle endurance in CKD. The decrease of absolute tension in the plantar flexor muscles in Nx rats was found to be due to muscle atrophy, as there was no decrease in specific muscle tension. Tamaki et al. (4) also reported that exercise endurance was decreased before a reduction in muscle power at early phase in 5/6 nephrectomized mice. The skeletal muscles in Nx rats had decreased muscle endurance in response to 20 or more repetitive electrical stimuli in both in vivo and ex vivo conditions. Such a detailed physiological phenotype of muscle endurance loss induced by CKD may provide clues to the underlying mechanisms. That is, the development of muscle fatigue induced by repeated short tetani in fast-twitch muscle fibers usually follows a characteristic pattern: an initial force decrease of ∼10% over ten tetani (*phase 1*), followed by a subacute period of slow force decline (*phase 2*), and finally a more rapid force decrease (*phase 3*) (31). Intriguingly, it has also been shown that the force decline during *phase 2* depends on mitochondrial dysfunction (32, 33). Therefore, the results of muscle physiological experiments in the present study suggest that decreased muscle endurance caused by CKD is mainly associated with mitochondrial dysfunction.

Muscle mass was reduced without myofiber-type transition in our CKD rat model. However, Acevedo et al. (34) reported that a myofiber-type transition from slow- to fast muscle fibers was observed in the tibialis anterior muscle 12 wk after the operative procedure for 5/6 nephrectomy. Considering that the present study was conducted 8 wk postoperatively, muscle endurance might be decreased before myofiber-type transition in CKD. In addition, it has been reported that disuse-induced muscle weakness is characterized by atrophy of MHC type I fibers with conversion from slow- to fast muscle fibers (35). The present study revealed that CKD can deteriorate muscle mass and endurance without the occurrence of myofiber-type transition.

Since the muscle physiological experiments indicated that mitochondrial dysfunction was associated with decreased muscle endurance in Nx rats, we assessed mitochondrial respiratory capacity and expression levels of subunits of respiratory chain complexes. Similar to the results of previous studies showing that mitochondrial OXPHOS is impaired in the skeletal muscle of CKD (4, 9, 36), the rate of oxygen consumption by electron transfer through mitochondrial respiratory complexes I-III-IV was impaired in Nx rats in the present study. This finding can be supported by the result that the expression level of protein constituting mitochondrial complex III (UQCRC2) in Nx rats was decreased in this study. Wang et al. (37) reported that expression levels of all complexes were decreased in rats 24 wk after 5/6 nephrectomy. Thome et al. (10) also reported that uremic metabolites significantly downregulated complexes III and IV, whereas Thome et al. (9) showed that reductions of mitochondrial respiratory complexes in mice with adenine-induced CKD did not reach statistical significance. The results of the present study taken together with the results of those previous studies indicate that complex III in the mitochondrial OXPHOS may be preferentially affected by CKD and may be a possible therapeutic target for CKD-related cachexia. However, these results only capture a portion of mitochondrial dysfunction and do not comprehensively elucidate how the energy-producing system, including mitochondria, is actually impaired by CKD during endurance exercise.

This study also revealed how the presence of CKD affected overall muscle energy metabolism during muscle fatigue induced by electrical stimulation. TCA cycle intermediates are known to be increased during exercise and the increase has been reported to be primarily mediated by large changes in succinate, malate, and fumarate (38). Increases of phosphoribosyl pyrophosphate, IMP, and inosine are induced by the consumption of high-energy phosphates of ATP and ADP or activation of the pentose phosphate pathway, shown in pre- and post-high-intensity exercise studies using horses (15). Indeed, in the PCA in our study, these substances were selected as principal determinants of resting and fatigued states in Sham rats, suggesting that the intensities of loads by electrical stimulation in this study were relatively high. On the other hand, metabolites in anaerobic glycolysis were the major determinants in Nx rats. Furthermore, the magnitude of increase in acetyl-CoA from a resting state to a fatigued state in Nx rats was less than that in Sham rats. Thus, these findings indicate that the metabolic flux from glycolysis to the TCA cycle, mainly acetyl-CoA, is inhibited in the skeletal muscle of CKD.

The major streams supplying acetyl-CoA are pyruvate, fatty acids, and amino acids and cellular NADH/NAD^+^ ratio is closely related with acetyl-CoA metabolism. In the present study, muscle fatigue significantly increased the NADH/NAD^+^ ratio in Sham rats, but such increase was not observed in Nx rats. Considering that PDH catalyzes the oxidative decarboxylation of pyruvate to produce NADH from NAD^+^ (39), changes in PDH activity may explain the differences in responsiveness to muscle fatigue between Sham and Nx rats. Interestingly, however, the activity of PDH was not reduced in Nx rats in our results. Based on the lack of change in pyruvic acid levels before and after fatigue and the results that PDH activity was not impaired, it is likely that the supply of acetyl-CoA from pyruvic acid is not impaired. Thome et al. (9) have shown that mice with adenine-induced CKD had decreased activities of pyruvate dehydrogenase (PDH), α-ketoglutarate dehydrogenase (AKGDH), and branched-chain α-keto acid dehydrogenase (BCKDH), whereas the other study showed that uremic toxins inhibited the activity of malate dehydrogenases (MDH) (10) and the study using 5/6 nephrectomized mice did not show the decreased activity of PDH without dietary protein (4). These results suggest that inactivation of PDH does not necessarily occur in renal failure and that other factors supplying acetyl CoA should be disrupted in CKD-related cachexia. AKGDH catalyzes the reaction of 2-oxoglutaric acid to succinyl-CoA. According to the finding of similar increases in succinic acid in Nx rats and Sham rats in this study, it is unlikely that AKGDH is markedly inhibited in response to fatigue in CKD. BCKDH regulates the degradation reaction from a branched-chain amino acid (BCAA) (40). The present study showed that the amounts of BCAAs including valine, leucine, and isoleucine were not different between resting and fatigued state in both Sham rats and Nx rats, suggesting that a change of BCKDH activity is not essential in enhanced muscle fatigue induced by high-intensity electrical stimulation for a short duration in CKD. Changes in malic acid levels during fatigue showed the same trend in Sham rats and Nx rats, but the trend for reduction in citric acid levels in response to fatigue in Sham rats was not observed in Nx rats. Therefore, the activity of MDH, which catalyzes the interconversion of malic acid and oxaloacetic acid, may be involved in the dysregulation of the TCA cycle during fatigue in CKD. Taken together, these findings indicate that dysfunction of several matrix dehydrogenases in the TCA cycle may complementarily affect decreased muscle endurance in CKD.

Although the details of fatty acid metabolism were not evaluated in the present study, it has recently been suggested that fatty acid oxidation also contributes to the energy supply during high-intensity exercise (41). Malonyl CoA has been reported to be one of the elements that regulate the transfer of fatty acids into mitochondria for fatty acid oxidation during exercise (42). In the present study, despite the significant decrease in carnitine in Nx rats in this study, the rate of decrease in malonyl CoA in Nx rats was significantly less than that in Sham rats. On the other hand, since malonyl CoA is catalyzed to acetyl-CoA by malonyl CoA decarboxylase (43), the reduced utilization of malonyl CoA may also contribute to the insufficient increase in acetyl CoA during fatigue in CKD.

The strength of this study is that it reproduces the phenomenon of fatigue in actual skeletal muscle itself, and the kinetics of metabolites in CKD during the fatigue can be compared with that in a Sham condition, a non-CKD condition. On the other hand, we acknowledge that the present study has several limitations. First, the present study focused primarily on changes in fast-twitch muscles in CKD-related cachexia. Indeed, fast-twitch muscles are responsible for a high percentage of muscle endurance during repeated muscle contractions of a few seconds, such as a fall during walking, and it has been shown that balance during walking is significantly impaired in lower limbs fatigued mainly by fast-twitch muscles (44, 45). Assessing the effects of CKD on slow-twitch muscles may differ from fast-twitch muscles in muscle physiology and metabolic phenotype. However, in slow-twitch muscles, a decrease in exerted tension occurs only after a muscle contraction is sustained for several minutes or longer, and we have confirmed this with ex vivo force analysis using slow-twitch soleus muscles (Supplemental Fig. S1). Second, the sample size for metabolomic analysis was small. However, it should be noted that the fatigue state was quantitatively induced by electrical stimulation in the present study. In fact, the four groups of Sham-Rest, Sham-Fatigue, Nx-Rest, and Nx-Fatigue were clearly separated in the PCA of results of metabolome analysis, suggesting that the sample size was at least sufficient to detect significant metabolites that are associated with CKD and a fatigue state. Finally, we only evaluated the activity of the PDH enzyme as one of the possible responsible mechanisms in the present study since numerous metabolic enzymes may be associated with metabolic phenotypes that were observed in the present study. Elucidating the causal relationship and mechanisms of metabolic changes in response to muscle fatigue in CKD are our future projects. For the similar reason, animal models of gene-editing or pharmacological intervention to determine causal relationships between CKD and decreased muscle endurance were not used in the present study. Because of the multifactorial nature of the pathophysiology of CKD-related cachexia, intervention using only a specific substance may capture a phenomenon that differs from the true complexity of the pathophysiology. Instead, since our previous study showed improvements in mitochondrial function and muscle endurance with high-intensity interval training (16), a study exploring optimal exercise or training interventions that improve the metabolic derangement and mitochondrial dysfunction induced by CKD should be performed in the future.

### Conclusions

The present study indicated that CKD deteriorates skeletal muscle endurance without myofiber type transition in association with mitochondrial dysfunction and inadequate supply of acetyl-CoA during muscle fatigue. The results of detailed physiological assessments and multi-omics metabolic analyses of skeletal muscle in a CKD model may contribute to the establishment of a therapeutic approach for CKD-related cachexia.

## DATA AVAILABILITY

Data will be made available upon reasonable request.

## SUPPLEMENTAL DATA

10.6084/m9.figshare.23661795 Supplemental Figs. S1 and S2: https://doi.org/10.6084/m9.figshare.23661795.

## GRANTS

This work was supported by Japan Society for the Promotion of Science (JSPS) KAKENHI Grant Numbers JP19K08522, JP20K18069, and JP23K10505.

## DISCLOSURES

No conflicts of interest, financial or otherwise, are declared by the authors.

## AUTHOR CONTRIBUTIONS

H.F., T.S., T.Y., N.I., Y.T., I.O., A.T., and N.T. conceived and designed research; H.F., T.S., Y.A., I.K., A.N., N.T., and N.Y. performed experiments; H.F., T.S., Y.A., and I.K. analyzed data; H.F., T.S., T.Y., N.T., interpreted results of experiments; H.F. prepared figures; H.F. drafted manuscript; H.F. and N.T. edited and revised manuscript; H.F., T.S., T.Y., N.I., Y.T., I.O., A.T., T.Y., and N.T. approved final version of manuscript.
